# Aldolase positively regulates of the canonical Wnt signaling pathway

**DOI:** 10.1186/1476-4598-13-164

**Published:** 2014-07-04

**Authors:** Michal Caspi, Gili Perry, Nir Skalka, Shilhav Meisel, Anastasia Firsow, Maayan Amit, Rina Rosin-Arbesfeld

**Affiliations:** 1 Department of Clinical Microbiology and Immunology, Sackler Faculty School of Medicine, Tel Aviv University, Ramat-Aviv, Tel Aviv 69978, Israel

**Keywords:** Wnt signaling, Aldolase, β-catenin, GSK-3β

## Abstract

The Wnt signaling pathway is an evolutionary conserved system, having pivotal roles during animal development. When over-activated, this signaling pathway is involved in cancer initiation and progression. The canonical Wnt pathway regulates the stability of β-catenin primarily by a destruction complex containing a number of different proteins, including Glycogen synthase kinase 3β (GSK-3β) and Axin, that promote proteasomal degradation of β-catenin. As this signaling cascade is modified by various proteins, novel screens aimed at identifying new Wnt signaling regulators were conducted in our laboratory. One of the different genes that were identified as Wnt signaling activators was Aldolase C (ALDOC). Here we report that ALDOC, Aldolase A (ALDOA) and Aldolase B (ALDOB) activate Wnt signaling in a GSK-3β-dependent mechanism, by disrupting the GSK-3β-Axin interaction and targeting Axin to the dishevelled (Dvl)-induced signalosomes that positively regulate the Wnt pathway thus placing the Aldolase proteins as novel Wnt signaling regulators.

## Introduction

One of the fundamental tasks of a cell in order to control its fate and the function of the entire organism is to create dynamic systems of signaling pathways. Today, it is well accepted that a few signaling pathways control the major developmental processes. When aberrantly regulated theses pathways lead to devastating diseases ranging from neurological diseases to cancer. One such pathway, which when up-regulated is implicated in a growing list of degenerative diseases and in most cases of colorectal cancer (CRC) is the Wnt signaling pathway [1]. In un-stimulated cell, the Wnt signaling cascade is silenced due to the activity of a dedicated cytoplasmic destruction complex that phosphorylates β-catenin, the key effector of the canonical Wnt pathway, marking it for ubiquitination, and subsequent degradation. This destruction complex consists of the scaffold protein Axin, the tumor suppressor adenomatous polyposis coli (APC) and the kinases glycogen synthase kinase-3 (GSK-3) α/β and casein kinase-1α (CKIα) [2,3]. The Wnt signaling cascade initiates with binding of the Wnt ligand to its receptor frizzled (Fz) and co-receptor low-density lipoprotein receptor-related protein 5/6 (LRP5/6). This event ultimately leads to accumulation and nuclear translocation of β-catenin resulting in expression of Wnt target genes [4]. Still, the mechanism of the Wnt signal transmission remains incompletely understood. According to the current model, the activated Wnt receptors recruit dishevelled (Dvl) to the plasma membrane. In turn, Dvl along with other Wnt signaling regulators such as LRP induce the formation of “puncta-like” structures classified as LRP-signalosomes [5]. In the signalosomes LRP is phosphorylated resulting in inhibition of GSK-3β which leads to the “β-catenin destruction complex” inactivation and accumulation of β-catenin. However this model is still being challenged and new Wnt signaling components and mechanisms of action are frequently being described. In an attempt to identify new Wnt signaling components we utilized a novel screening technique based on expression of an episomal cDNA library in mammalian cells followed by selection of clones that survive only in the continuous presence of Wnt stimulus [6]. One of the genes that were isolated in three separate experiments was Aldolase C fructose bisphosphate (ALDOC) the fourth enzyme of glycolysis, which catalyzes reversible cleavage of fructose 1,6-bisphosphate into glyceraldehyde 3-phosphate and dihydroxyacetone phosphate [7]. In vertebrates, the Aldolase family consists of three isozymes that are structurally very similar: Aldolase A (ALDOA), the muscle and red blood cells isoform; Aldolase B (ALDOB), the liver, kidney and intestine isoform; and ALDOC, the brain and nervous system isoform [8,9]. Although the role of Aldolase in metabolism is well established, there is growing evidence for many alternative functions for this enzyme. In particular, Aldolase interacts with various proteins unrelated to glycolytic enzymes, including cytoskeleton proteins such as F-actin [10,11], WASP [12] and tubulin [13]. Aldolase also interacts with other types of proteins such as proteins involved in vesicle and intracellular trafficking [14-16] proton pumps [16,17] and is crucial for proliferation of cancer cells through a non-glycolytic pathway [18].

In the present study we show that Aldolase activates Wnt signaling by forming a complex with GSK-3β that disrupts the GSK-3β-Axin interaction leading to membrane translocation of Axin. These findings indicate that Aldolase isomers can function as novel regulators of the canonical, oncogenic Wnt signaling pathway and may become new anti-cancer therapeutic targets.

## Materials and methods

### Cell culture and transfection

Human embryonic kidney 293T (HEK293T), human cervical cancer (HeLa), monkey kidney (COS-7) and the human colon carcinoma SW480 cell lines were maintained in Dulbecco’s modified Eagle’s medium (GIBCO) supplemented with 10% fetal bovine serum and 100 units/ml penicillin/streptomycin (Beit-Haemek). Cells were cultured at 37°C in a humidified incubator with 5% CO_2_. For HEK293T cells, transfections were carried out using the standard CaPO_4_ precipitation method, or using Polyethylenimine (PEI) reagent (Polysciences) following manufacturer’s guidelines. For HeLa, COS-7 and SW480 cells, Polyethylenimine (PEI) reagent (Polysciences) was used. SB (SB-216763) is a small molecule that competes with ATP and potently inhibits the activity of GSK-3β was used (Sigma, Israel; 10 μM; 4 hours).

### Plasmids

GFP-ALDOB expression vector was constructed by inserting ALDOB cDNA (provided by Marçal Pastor-Anglada, Universitat de Barcelona, Barcelona, Spain) into pEGFP-C2 (Clontech, Palo Alto, CA) using *Eco*RI and *Sal*I restriction sites. GFP-ALDOC was constructed in our laboratory by amplifying ALDOC cDNA by PCR (using the primers 5′-AGAGGGATCCGCAT GCCTCACTCGTACCCAGCC-3′ and 5′- AGAGCTCGAGGTAGGCATGGTTG GCAATGTAGAG-3′) and subcloning into pEGFP-C2 using *Bgl*II and *Sal*I restriction sites. The ORF of human Aldolase A was cloned into pEGFP-C2 vector using EcoRI and KpnI sites. For PCR we used the primers: EcoRI-AldA-Fw- AAAAGAATTCATGCCCTACCAATATCCAGC, and KpnI-AldA-Rv-AAAGGTACCATAGGCGTGGTTAGAGACGAAG. HA-GSK-3β and FLAG–GSK-3β expression vectors were kindly provided by T.C. Dale (Developmental Biology, Chester Beatty Laboratories, Institute of Cancer Research, London, UK) and Hagit Eldar-Finkelman (Tel-Aviv University, Tel-Aviv, Israel), respectively. GFP-Axin and FLAG-Axin expression vectors were kindly provided by Mariann Bienz (MRC Laboratory of Molecular Biology, Hills Road, Cambridge, UK) and T.C. Dale, respectively, and were described previously. The Wnt-responsive TCF-dependent luciferase constructs pTOPFLASH and its mutated version pFOPFLASH were kindly provided by H. Clevers (Center for Biomedical Genetics, Utrecht, Netherlands) and were described before. pSV-β-Galactosidase Control Vector and pCMV-Renilla were purchased from Promega (Madison, WI).

### Luciferase reporter assay

Twenty four hours after seeding in 24-well plates at 1×10^5^ cells per well, cells were transfected with relevant DNA plasmids, along with pGL3-OT (pTOPFLASH) or pGL3-OF (pFOPFLASH) – luciferase reporter constructs. These constructs contain the firefly luciferase open reading frame under the control of three copies of either wild-type (pTOPFLASH) or mutated TCF binding element (pFOPFLASH) [19]. These constructs are used for assessing changes in the canonical Wnt pathway. The β-galactosidase construct (in HEK293T cells) or CMV-Renilla (in SW480 cells) were used to monitor transfection efficiency. Forty eight hours post-transfection, cells were washed with phosphate-buffered saline (PBS) and harvested on ice using Reporter Lysis Buffer (Promega). Cell lysates were centrifuged for 15 minutes at 14,000 rpm at 4°C and their luciferase activity was measured following manufacturer’s instructions. Specificity of luciferase activity was validated using the pFOPFLASH plasmid. Residues of supernatants were analyzed by Western blotting as described below.

### Western blot analysis and immunoprecipitation

HEK293T cells were transfected as indicated above, and 48 hours later washed with PBS and harvested on ice using lysis buffer (50 mM Tris pH 7.5, 150 mM NaCl, 1% Triton), or radioimmunoprecipitation assay (RIPA) buffer (50 mM Tris-HCl pH 7.4, 1% NP-40, 0.25% sodium deoxycholate, 150 mM NaCl, 1 mM EDTA) supplemented with 1% protease inhibitor cocktail (Sigma). Cell lysates were centrifuged for 15 minutes at 10,000-14,000 rpm at 4°C. Supernatants were separated on 7.5% or 10% SDS-polyacrylamide gel electrophoresis (SDS-PAGE), and proteins were transferred to nitrocellulose membranes. After blocking with 5% low fat milk, membranes were incubated with primary antibodies, washed three times with 0.001% tween-20 in PBS, incubated for 60 minutes with secondary antibodies, washed again three times and exposed to enhanced chemiluminescence (ECL) detection analysis using horseradish peroxidase-conjugated secondary antibodies.

For immunoprecipitation (IP) assays, cell lysates were incubated following centrifugation with anti-FLAG M2-agarose affinity gel (Sigma), with rotation for two hours at 4°C. Alternatively, cell lysates were incubated with the specific antibody for two hours on ice prior to two hours rotated incubation with protein A/G agarose (Santa Cruz Biotechnology) at 4°C. Following incubation, beads were collected by slow centrifugation, washed four times with lysis buffer and analyzed by Western blotting as described. For endogenic IP assays, mouse brain extracts were homogenized in RIPA buffer supplemented with 1% protease inhibitor cocktail. Following centrifugation, supernatants were incubated for two hours on ice with the relevant antibody or with control unimmuned serum, and then incubated at 4°C with rotation with protein A/G agarose and separated by SDS-PAGE as designated before. The following antibodies were used (for immunoblotting, unless mentioned otherwise): goat anti-Aldolase B (1:500; Santa Cruz Biotechnology), goat anti-Aldolase C (1:500; Santa Cruz Biotechnology), goat anti-Axin (1:250 for IP; Santa Cruz Biotechnology), rabbit anti-SOX-9 (1:500; Millipore) rabbit anti-GFP (1:1000; Santa Cruz Biotechnology), mouse anti-GFP (1:1000; Abcam), mouse anti-GSK-3β (1:5000; BD Transduction Laboratories), rat anti-HA (1:5000; Roche), mouse anti-FLAG (1:5000; Sigma), mouse anti-β-catenin (1:100; BD Transduction Laboratories), mouse anti-β-catenin active (1:1000; Millipore), rabbit anti-phospho-β-catenin (1:1000; cell signaling), and mouse anti-Tubulin (1:10,000; Sigma), anti-Striatin (1:1000; BD Transduction Laboratories), Rabbit anti-GSK-3β used for IP was kindly provided by Hagit Eldar-Finkelman (Tel-Aviv University, Tel-Aviv, Israel). Anti-goat horseradish peroxidase-conjugated secondary antibody was obtained from Santa Cruz Biotechnology and was used at a 1:5000 dilution. Anti-mouse and anti-rabbit secondary antibodies were obtained from Jackson Immuno Research and were used at a 1:10,000 dilution.

### Immunufluorescence

HEK293T, HeLa or COS-7 cells were seeded on glass coverslips in 24-well plates and transfected as mentioned previously. Forty eight hours after transfection, cells were washed with PBS and fixed in PBS containing 4% paraformaldehyde (PFA) for 20 minutes. Fixed cells were washed twice with PBS, permeabilized with PBS containing 0.1% Triton (PBT) for 10 minutes and blocked in PBS containing 1% BSA and 0.1% Triton (BBT) for one hour. Afterwards, cells were incubated at room temperature with primary antibodies for 60 minutes, washed three times with PBT, incubated with secondary antibodies for 30 minutes, and washed again three times. Finally, cell nuclei were stained with 10 μg/ml 4′,6-Diamidino-2-phenylindole (DAPI, Sigma) for 5 minutes. Slides were visualized by confocal microscopy or by phase contrast microscopy (Leica SP2, Leica Microsystems, Bannockburn, IL).

The following antibodies were used: goat anti-Aldolase B (1:100; Santa Cruz Biotechnology), goat anti-Aldolase C (1:100; Santa Cruz Biotechnology), rabbit anti-FLAG (1:400; Sigma), mouse anti-GSK-3β (1:300; BD Transduction Laboratories), rat anti-HA (1:300; Roche), mouse anti-myc (1:200; Santa Cruz Biotechnology). Anti-goat, anti-mouse, anti-rabbit and anti-rat fluorescent antibodies were obtained from Invitrogen and were used at a 1:500 dilution.

### siRNA assay

HEK293T cells were transfected with 30nM GSK siRNA (siGENOME SMART pool M-003010-03-0005 hGSK3b NM-002093), 50 or 100nM siGENOME ALDOC siRNA; NM_005165; M-012697-01-0005 or non-targeting RNA oligonucleotides as scrRNA, using DharmaFECT-1 as transfection reagent; siRNA and scrRNA oligonucleotides, together with the mentioned reagent, were all purchased from Thermo Scientific Dharmacon (Essex, UK). Cells were either harvested for western blot analysis after 72 h or transfected with the relevant DNA plasmids after 24 h. Forty eight h later the transfectedcells were harvested and analyzed using Western blots as described above All animal work was conducted according to national and international guidelines and approved by the Tel Aviv University review board.

## Results

### Aldolase isomers activate the canonical Wnt signaling pathway

**Figure 1:**
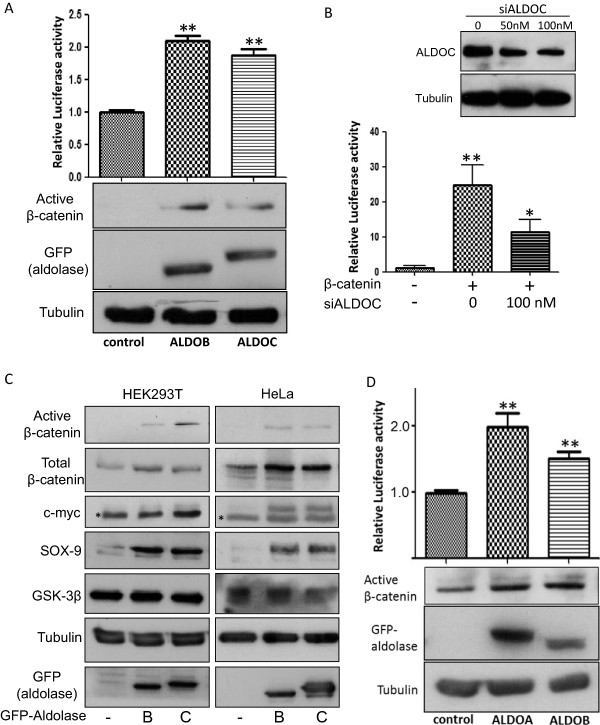
**Aldolase isomers activate the canonical Wnt signaling pathway. (A)** HEK293T cells were co-transfected with GFP-ALDOC, GFP-ALDOB, an empty vector, pTOPFLASH or pFOPFLASH and β-gal. Forty eight hours later, cells were harvested and lysates were measured for their luciferase activity, (upper panel) and levels of endogenous active β-catenin (lower panel). Anti-GFP antibody was used to detect Aldolase proteins. Data presented describe relative mean values and standard deviations of four independent experiments performed in duplicates. Asterisks indicate significant difference in Wnt signaling levels (** p < 0.01, Student’s *t* test). **(B)** HEK293T cells were transfected with different concentrations of siALDOC RNA. Seventy-two hours later cells were harvested and levels of endogenous ALDOC were determined (upper panel). Alternatively, HEK293T cells were co-transfected with GFP-β-catenin or an empty vector along with the pTOPFLASH or pFOPFLASH and β-gal, and 24 h later the cells were transfected with siALDOC (100 mM). Forty eight hours later the cells lysates were measured for their luciferase activity (lower panel). Asterisks indicate significant difference in Wnt signaling levels (* p < 0.05, ** p < 0.01, Student’s *t* test). **(C)** HEK293T or HeLa cells were transfected with the two Aldolase isomers as indicated. Forty eight hours later, lysates were subjected to western blot analysis using specific antibodies to distinct members of the Wnt signaling pathway. An anti-GFP antibody was used to detect Aldolase. Asterisk points to a non-specific band. **(D)** HEK293T cells were co-transfected with GFP-ALDOA, GFP-ALDOB or an empty vector, pTOPFLASH or pFOPFLASH and β-gal. Forty eight hours later, cells were harvested and lysates were measured for their luciferase activity, (upper panel) and levels of endogenous active β-catenin (lower panel). Data presented describe relative mean values and standard deviations of 3 independent experiments performed in duplicates or triplicates. Asterisks indicate significant difference in Wnt signaling levels (** p < 0.01, Student’s *t* test). Tubulin served as a loading control in all experiments.

ALDOC was isolated in a screen aimed at identifying new Wnt signaling activators [6]. To validate this effect, both ALDOC and ALDOB, were tested for their ability to activate Wnt signaling. Results show that ectopic expression of both proteins increase Wnt/β-catenin mediated transcription (Figure 1A, upper panel) and more importantly led to increased levels of the endogenous active form of β-catenin (Figure 1A, lower panel). Similarly, reducing the endogenous levels of ALDOC by specific siRNA oligonucleotides led to decreased Wnt/β-catenin mediated transcription (Figure 1B). Activation of the Wnt cascade ultimately results in elevated levels of Wnt target genes. Our results show that expression of both ALDOC and ALDOB dramatically induced the expression of Wnt target genes such as c-myc and SOX-9 in addition to increasing the levels of active and total β-catenin in both HEK293T and HeLa cells (Figure 1C; Additional file 1: Figure S1). The widely expressed ALDOA that is highly similar to ALDOB was also tested. Our results demonstrate that similarly to the other ALDO isoforms, expression of ALDOA leads to enhanced expression of active β-catenin and activates the canonical Wnt signaling cascade (Figure 1D). When examining the ALDO activity of our constructs (Additional file 2) we noticed that all our ALDO isomers have similar activity (Additional file 1: Figure S2A) although the activity of ALDOA is known to be significantly higher than that of the other ALDO isomers [20,21]. However, the differences in activity between ALDOA and ALDOB are so extensive when the activity of the purified proteins is compared (around 6 fold). In contrast, when the ALDO activity of crude extracts from bacteria overexpressing ALDOB and ALDOA are measured the differences are reduced to two folds [21]. In addition this study also shows that although Rabbit muscle ALDOA has very similar activity to recombinant ALDOA, the activity of the recombinant ALDOB is much higher than that of Human liver ALDOB and reached the range of ALDOA [21]. Taking these results together, we speculate that the different ALDO isomers utilized in our current study show similar activity due to the fact that crude mammalian cell extracts ectopically expressing recombinant ALDO isomers were used.

### Aldolase activation of the Wnt pathway depends on an intact “β-catenin degradation complex”

**Figure 2:**
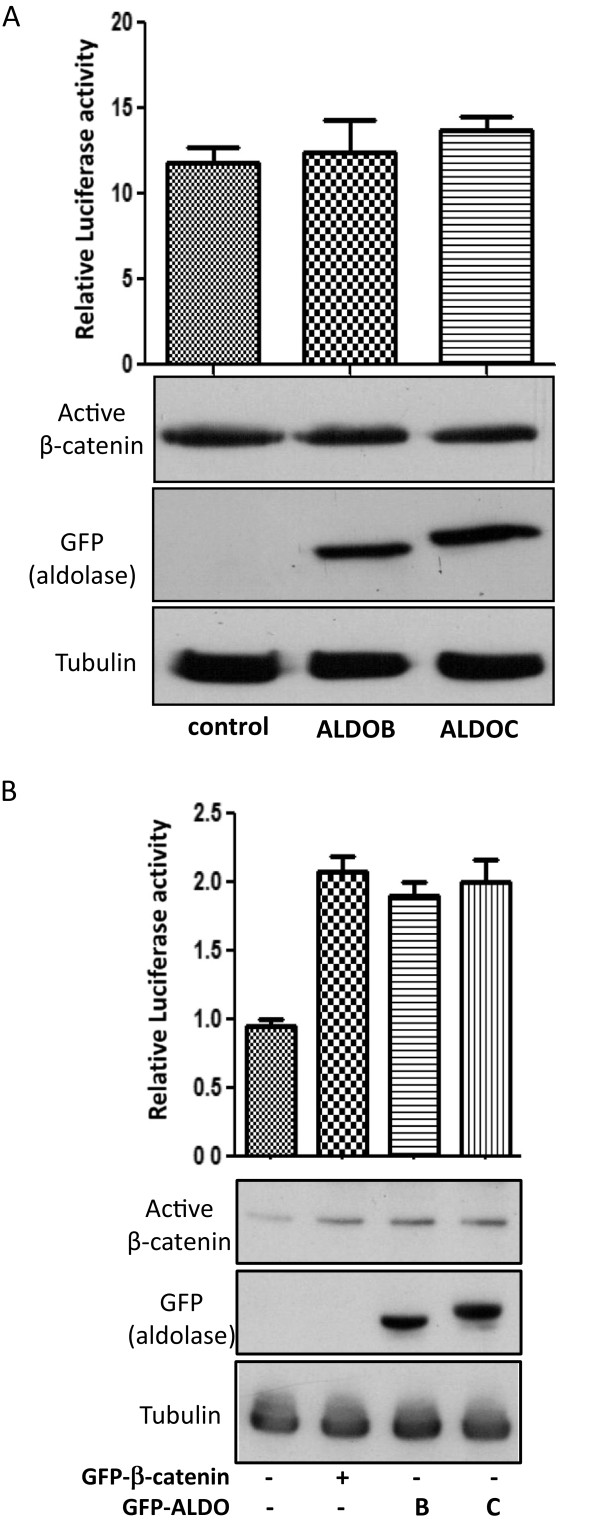
**Aldolase activation of the Wnt pathway depends on the “β-catenin degradation complex”. (A)** SW480 cells were co-transfected with the following plasmids: GFP-ALDOC, GFP-ALDOB or an empty vector, pTOPFLASH or pFOPFLASH and Renilla. Forty eight hours later, cells were harvested and lysates were measured for their luciferase activity, (upper panel) and levels of endogenous active β-catenin by western blot (lower panel). Tubulin served as a loading control. Data presented describe relative mean values and standard deviations of four independent experiments performed in duplicates or triplicates. **(B)** HEK293T cells were transfected with GFP, GFP-β-catenin, GFP-ALDOC or the GFP-ALDOB constructs as indicated, along with the pTOPFLASH or pFOPFLASH and β-gal plasmids. Luciferase assay (upper panel) and western blot analysis (lower panel) were performed as described.

To examine whether ALDOB and ALDOC activity requires an intact destruction complex, SW480 cells were used. In these cells the APC protein is mutated [22] and as a result the “β-catenin degradation complex” is not functional. Results show that both ALDOB and ALDOC had no effect on Wnt/β-catenin mediated transcription or β-catenin protein levels in these cells (Figure 2A) thus suggesting that the destruction complex may be required for the activity of Aldolase.

### GSK-3β interacts with Aldolase proteins

**Figure 3:**
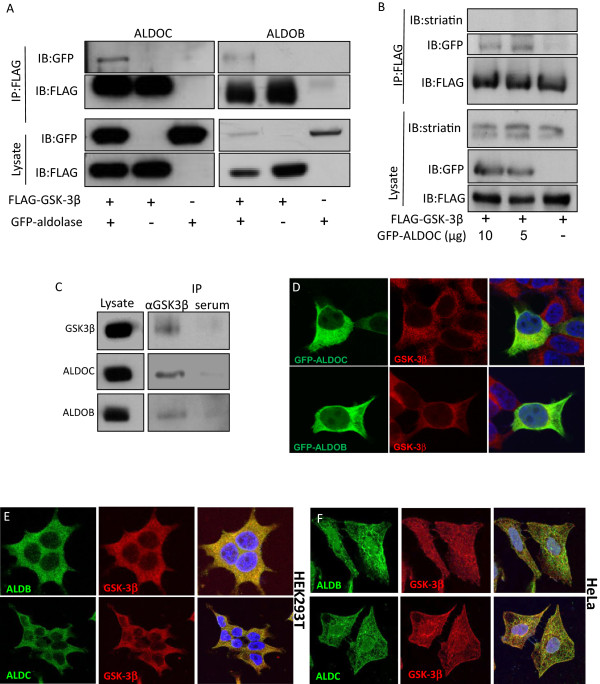
**GSK-3β co-localizes and interacts with Aldolase proteins. (A)** HEK293T cells were co-transfected with FLAG-GSK-3β or an empty vector, and with GFP-ALDOC (left), GFP-ALDOB (right) as indicated. Forty eight hours post transfection, cells were harvested and lysates were co-immunoprecipitated with anti-FLAG M2 beads, separated by SDS-PAGE and visualized using the indicated antibodies. **(B)** HEK293T cells were co-transfected with FLAG-GSK-3β and increasing concentrations of GFP-ALDOC (0-10 mg DNA). Forty eight hours post transfection, cells were harvested and lysates were co-immunoprecipitated with anti-FLAG M2 beads, separated by SDS-PAGE and visualized using the indicated antibodies. Striatin was used as a negative control to rule out non-specific interactions. **(C)** Mouse brain lysates were co-immunoprecipitated with rabbit anti-GSK-3β antibody or with control un-immuned serum incubated with protein A/G beads and separated by SDS-PAGE. Blots were incubated with anti-GSK-3β, anti-ALDOC or anti-ALDOB antibodies. **(D)** HEK293T cells grown on cover slips were transfected with GFP-ALDOC (upper panel) or GFP-ALDOB (lower panel). Forty eight hours later, cells were fixed, permeabilized, and incubated with an anti-GSK-3β antibody. Cell nuclei were then stained with DAPI, and viewed using confocal microscopy. **(E)** Untransfected HEK293T cells grown on cover slips were subjected to immunofluorescence assay as in **D**, using the indicated antibodies. **(F)** HeLa cells grown on cover slips were subjected to immunofluorescence assay as in **D**, using the indicated antibodies.

Both the two GSK-3 isoforms and the three Aldolase isozymes are metabolic enzymes. While GSK-3α/β inhibit glycogen synthase thus preventing the conversion of glucose to glycogen [23], the Aldolase proteins are responsible for the conversion of fructose 1,6-diphosphate into dihydroxyacetone phosphate (DHAP) and glyceraldehyde-3-phosphate (GA3P) [7]. Thus, we examined whether ALDOB and ALDOC interact with GSK-3β. HEK293T cells were co-transfected with plasmids encoding for FLAG-tagged GSK-3β and GFP-tagged ALDOB or ALDOC. As shown in Figure 3A, GSK-3β co-immunoprecipitated with the Aldolase proteins. Expressing different amounts of the ALDOC proteins did not alter the amount of the ALDOC-GSK-3β complex (Figure 3B). Importantly, endogenous GSK-3β specifically co-immunoprecipitated with both ALDOB and ALDOC in brain extracts (Figure 3C). Examining the subcellular localization of GSK-3β and Aldolase revealed that both ectopically expressed and endogenous ALDOB and ALDOC co-localize with endogenous GSK-3β in both 293T and HeLa cells (Figure 3D, E, F).

### Aldolase depends on GSK-3β for activating the Wnt pathway but does not affect the phosphorylation of β-catenin

**Figure 4:**
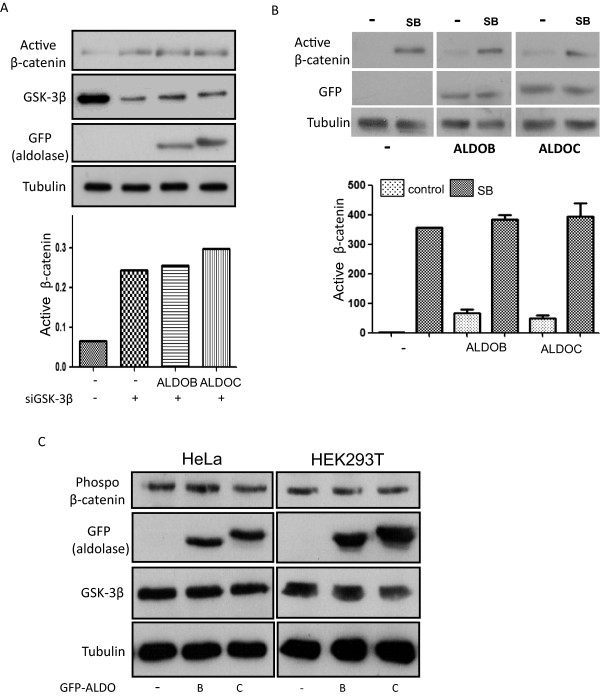
**Aldolase requires GSK-3β for activating the Wnt pathway but does not affect the phosphorylation of β-catenin. (A)** HEK293T cells were subjected to GSK-3β depletion using siGSK-3β RNA oligonucleotides. Twenty four hours later, cells were co-transfected with GFP-ALDOC, GFP-ALDOB or an empty vector. After additional forty eight hours, cells were harvested and separated by SDS-PAGE. Blots were incubated with anti-active β-catenin, anti-GFP, anti-GSK-3β or anti-tubulin as indicated (upper panel). TINA analysis of active β-catenin band intensity is presented (lower panel). **(B)** Untransfected or cells transfected with ALDOB or ALDOC were treated with SB (10 μM; 4 hours) as indicated. Twenty four hours later the cells were harvested and separated by SDS-PAGE. Blots were incubated with the indicated antibodies (upper panel). TINA analysis of active b-catenin band intensity was performed on 2 separate experiment (lower panel). **(C)** HEK293T or HeLa cells were transfected with GFP-ALDOC, GFP-ALDOB or an empty vector. Forty eight hours later, cells were harvested and lysates were subjected to western blot analysis using specific antibodies to anti-phospho-β-catenin, anti-GFP, anti-GSK-3β or anti-tubulin as indicated.

Next we examined whether Aldolase depends on GSK-3β for its activity in Wnt signaling. SiRNA oligonucleotides targeting GSK-3β were used to silence endogenous GSK-3β in HEK293T cells which, as expected, led to increased levels of active β-catenin. Importantly, depletion of GSK-3β hampered the ability of ALDOB and ALDOC to elevate the β-catenin protein levels (Figure 4A) as shown earlier (Figure 1). Similarly, inhibiting GSK-3β (and thus elevating the levels of active β-catenin) by using SB [24] abolished the activity of the Aldolase proteins on β-catenin (Figure 4B). As GSK-3β phosphorylates β-catenin, thus targeting the latter for degradation we examined whether expression of ALDOC and ALDOB change the phosphorylation levels of β-catenin. Results indicate that ALDOC and ALDOB do not affect the phosphorylation levels of β-catenin (Figure 4C).

### Aldolase activates Wnt signaling by disrupting the Axin-GSK-3β interaction and targeting Axin to the Dvl “puncta”

**Figure 5:**
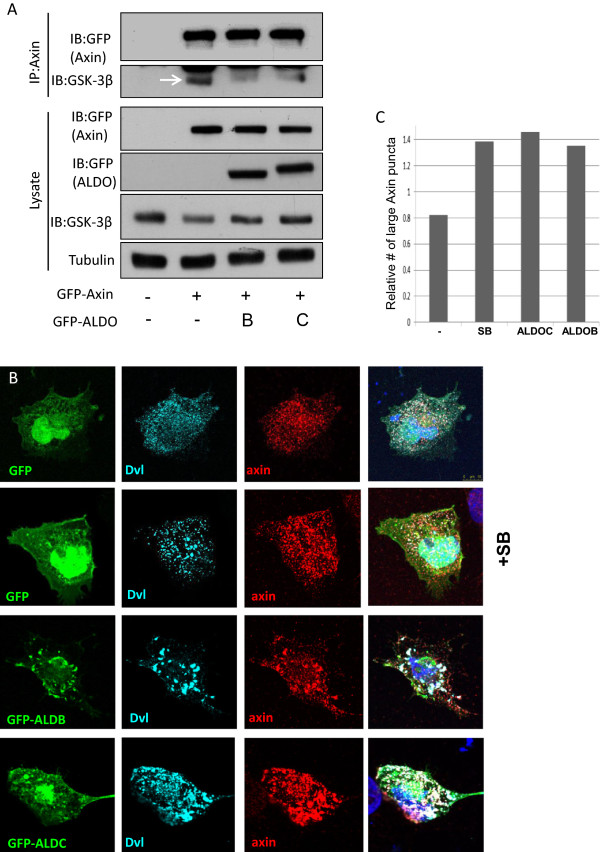
**Aldolase activates Wnt signaling by disrupting the Axin-GSK-3β interaction and targeting Axin to the Dvl “puncta”. (A)** HEK293T cells were co-transfected with GFP-Axin or an empty vector, and with GFP-ALDOC, GFP-ALDOB or an empty vector. Forty eight hours post transfection, cells were harvested and lysates were co- immunoprecipitated with goat anti-Axin antibody or with control un-immuned serum and separated by SDS-PAGE. Blots were incubated with anti-GFP; anti-GSK-3β or anti-tubulin **(B)** COS-7 cells were co-transfected with myc-Dvl and FLAG-Axin along with GFP-ALDOB, GFP-ALDOC or an empty vector. Forty eight hours post transfection; cells were fixed, permeabilized and incubated with anti-FLAG and anti-myc antibodies. Cell nuclei were then stained with DAPI, and visualized using a confocal microscopy. SB was used as a positive control for activation of the Wnt pathway **(C)** Stained cells from B were scored according to Axin distribution in large “puncta” (2 for only large, 1 for large and small, 0 small or diffused distribution) 20-25 cells were scored for each condition.

In the absence of a Wnt signal GSK-3β phosphorylates Axin which leads to enhanced activity of Axin and stabilization of the cytoplasmic “β-catenin degradation complex”. However, when the Wnt signal is activated, the “β-catenin degradation complex” disassembles and Axin is recruited to Dvl-induced “puncta” suggested to function as signalosomes [5,25]. Our results show that when over-expressed, both ALDOB and ALDOC disrupt GSK-3β-Axin interaction thus GSK levels detected in the complex are reduced (Figure 5A). Importantly, expression of the Aldolase proteins induce formation of large Dvl-Axin “puncta” that are similar to those seen when the specific GSK-3β inhibitor SB is used (Figure 5B-C).

## Discussion

**Figure 6:**
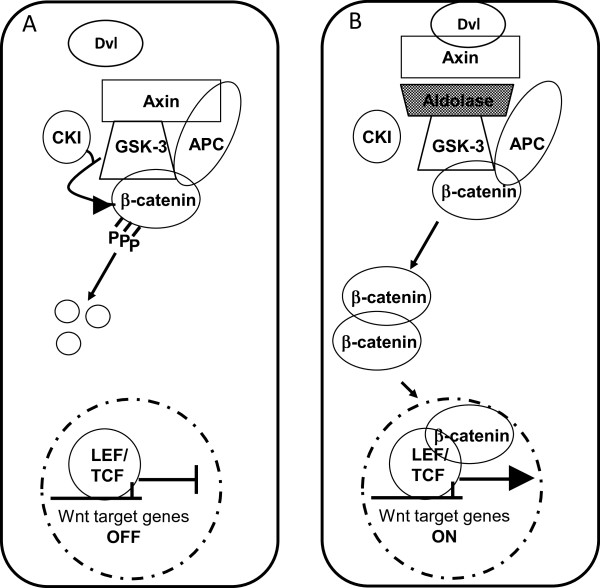
**A hypothetic model for the role of the Aldolase proteins in Wnt signaling stimulation. (A)** In the absence of Wnt stimulation, β-catenin is degraded by a dedicated cytoplasmic “β-catenin degradation protein complex” **(B)** in cells that express high levels of the Aldolase proteins, Aldolase binds GSK-3β, the “β-catenin degradation protein complex” is disassembled, disheveled and Axin re-localize to the membrane and the Wnt cascade is activated.

The canonical Wnt signaling pathway regulates the stability of the β-catenin protein. In the absence of a Wnt signal, β-catenin resides in a large cytoplasmic protein complex where it is phosphorylated and subsequently degraded. Activation of the Wnt cascade leads to inhibition of this β-catenin degradation complex in different mechanisms [26], some just recently proposed [27]. To further characterize and understand the Wnt cascade we have developed a novel screen aimed at identifying novel activators of the Wnt signaling pathway [6]. One of the genes isolated in this screen was ALDOC. Our results show that all three ALDO isoforms activate Wnt signaling; over-expression of each protein was sufficient to induce a significant increase of the endogenic signal, without ectopic activation (such as β-catenin or other known positive regulators of the pathway). Similarly, expression of the Aldolase proteins induced expression of several endogenous Wnt target genes. This may imply that Aldolase, primarily a glycolytic enzyme, could act as a colorectal oncogene – an assumption that correlates with the Warburg effect describing enhanced glucose uptake and glycolysis in cancerous cells [28]. Accordingly, over-expression of ALDOA and ALDOC were previously reported in various tumor types [29,30]. A recent paper demonstrated that ALDOA is critical for proliferation of transformed cell lines, though not by means of its glycolytic functions [18]. Moreover, ALDOA was lately reported as a prognostic marker of colorectal cancer progression, highly expressed in disease stages I and IV [31]. Nonetheless, it should be noted that ALDOB was found to be down-regulated in the progressive stages of hepatocellular carcinoma, probably due to transition of the cancerous cells into utilizing alternative paths for energy sources, for instance – ALDOA overexpression [32]. The transition of malignant tissues into over-expressing ALDOA at the expense of the prevalent Aldolase isozyme in the normal tissue was also reported, along with the decrease of serum ALDOB levels in malignant tissues, including in patients with gastric cancer [33]. It is important to note that there is a de-differentiation of tissue-specific expression to the embryonic pattern in both cancer and cell lines and that this is the major reason that Aldolase B and/or C give way to the embryonic Aldolase A in mammalian cells. Given the role of GSK-3β in regulation of carbohydrate metabolism, we hypothesized that GSK-3β might be the mediating factor for Aldolase – a glycolytic enzyme in its interaction with the degradation complex. Our results support this hypothesis, providing evidence of a physical interaction between GSK-3β and each of the Aldolase isozymes. Assuming that Aldolase activates the Wnt signaling pathway through an interaction with the β-catenin degradation complex and particularly GSK-3β, we speculated that neutralizing them would diminish the effect induced by Aldolase. Indeed, transfections of the human colon carcinoma cell line SW480, in which the destruction complex is inactive, failed to activate the Wnt cascade upon Aldolase overexpression. Furthermore, Aldolase over-expression could not stabilize the active form of β-catenin even when GSK-3β was specifically depleted using siRNA oligonucleotides. Together, these findings suggest that Aldolase interacts and requires GSK-3β for increasing Wnt signaling. Moreover, over-expression of ALDOB or ALDOC altered the expression pattern of Axin from cytoplasmic scattered punctate expression to accumulation along the plasma membrane. Similar changes in the expression pattern of Axin were previously reported. Relocation of Axin from scattered puncti to the cytoplasm was explained by a dynamic exchange of Axin between punctuate and cytoplasmic pools, leading to increased Wnt signaling [34,25,35]. Axin interacts with the other components of the β-catenin degradation complex, including GSK-3β, to create a scaffold for their activity [36]. GSK-3β does not just bind Axin, but also phosphorylates it to regulate its stability [37]. Hence, we propose that the cytoplasmic complex formed between Aldolase and GSK-3β, reduces the ability of the latter to bind Axin Figure 6. Indeed, our findings suggest a mechanism by which Aldolase may activate the Wnt pathway by inhibiting the affinity of GSK-3β to Axin, perhaps acting as a competitive inhibitor. This leads to destabilization of the “β-catenin destruction complex” and activation of β-catenin/TCF mediated transcription. Under conditions in which the β-catenin degradation complex is inactive, as in SW480 cells or upon depletion of GSK-3β, Aldolase cannot interfere with the activity of the complex and therefore cannot induce activation of Wnt signaling. We speculate that the relocation of Axin to the plasma membrane – which hinders the function of the degradation complex as well – is secondary to the disruption of the GSK-3β-Axin interaction, as Aldolase activity is GSK-3β-dependent; this relocation may provide further enhancement or stabilization of the primary effect induced by Aldolase.

The Wnt signaling pathway is modified by various proteins, some are known and others are yet to be revealed. The presented work introduces the role of ALDOB and ALDOC as positive regulators of the pathway characterizes their relationship with components of the Wnt cascade and proposes a mechanism for their action. As over-expression of Aldolase induces Wnt signaling, Aldolase might act as a colorectal oncogene, and therefore serve as a putative therapeutic target for cancer treatment or diagnosis.

## Competing interests

The authors declare that they have no competing interests.

## Authors’ contributions

MC participated in the study design. MC, GP, SM, NS and AF performed the experiments. MA designed and performed the cloning vectors. MC, GP and RRA wrote the paper. RRA supervised the entire project. All authors read and approved the final manuscript.

## Supplementary Material

Additional file 1: Figure S1 TINA analysis of three separated experiments where the effect of GFP, GFP-ALDOB and GFP-ALDOC over-expression on different components of the Wnt cascade was measured. A representative blot is shown in Figure 1C. Click here for file

Additional file 2 **Enzymatic activity of wild type and mutated aldolases.** HEK293T cells were transfected with the indicated plasmids and subjected to immunoprecipitation using mouse anti-GFP antibody. Enriched aldolase proteins were then incubated with Fructose-1,6-bisphosfate and Hydrazine sulfate for 90 min. in order to measure enzymatic activity. A. Activity of the different aldolase isoforms compared to non-transfected extracts. B. HEK293T cells lysates were harvested as described and subjected to Western blot analysis to detect the transfected ALDO proteins. C. Purified ALDX from rabbit muscle (Sigma) was used as positive control. Click here for file
