# Supplementary material for: Aldolase positively regulates of the canonical Wnt signaling pathway
Source: Mol Cancer. 2014 Jul 4;13:164. doi: 10.1186/1476-4598-13-164 (PMC4094682; doi:10.1186/1476-4598-13-164)
Supplement: Additional file 1: Figure S1 — TINA analysis of three separated experiments where the effect of GFP, GFP-ALDOB and GFP-ALDOC over-expression on different components of the Wnt cascade was measured. A representative blot is shown in Figure 1C. [file 1476-4598-13-164-S1.pptx]

## Slide 1
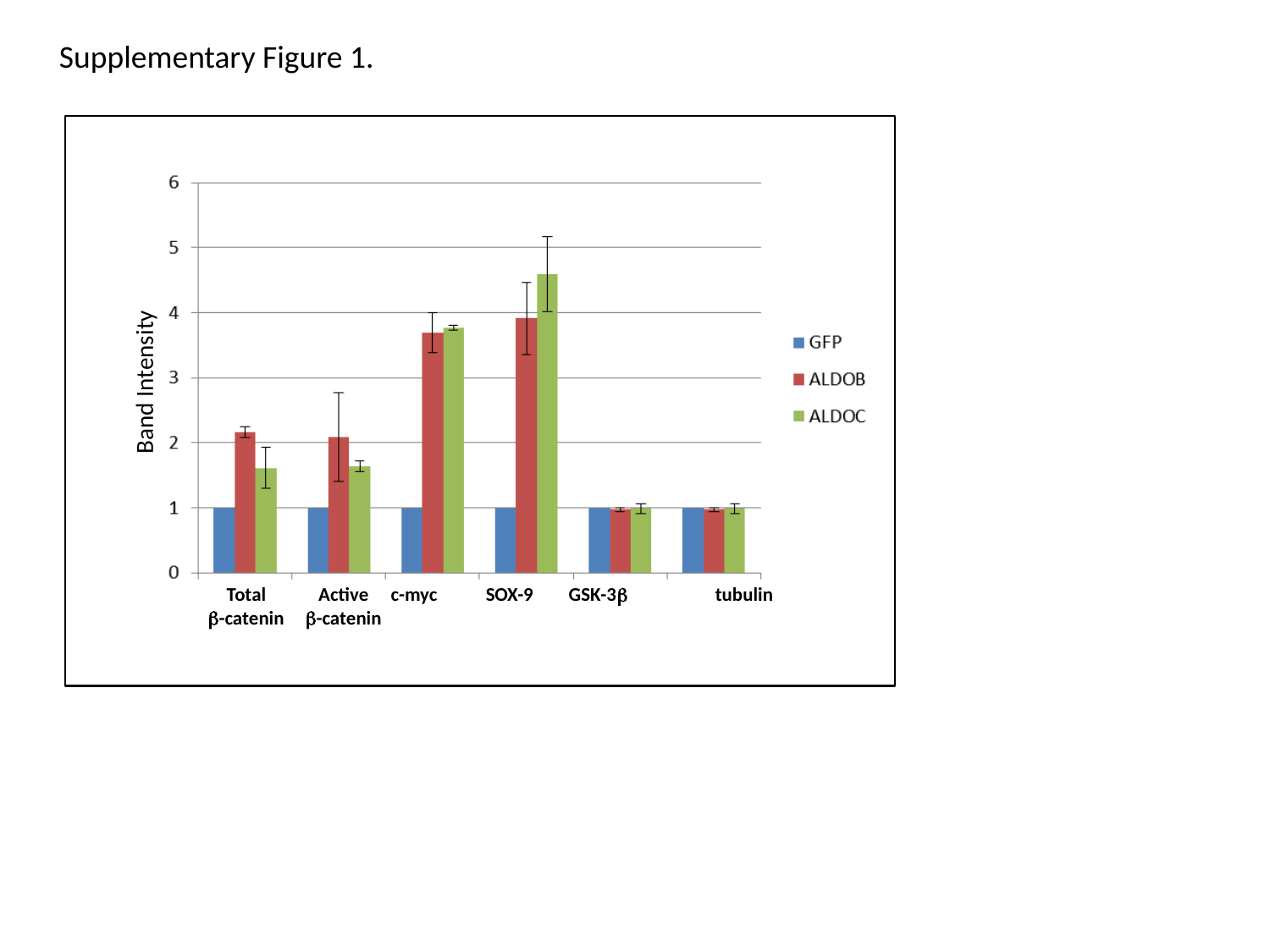

Supplementary Figure 1.
Band Intensity
Total
b-catenin
Active
b-catenin
c-myc SOX-9 GSK-3b tubulin

## Slide 2
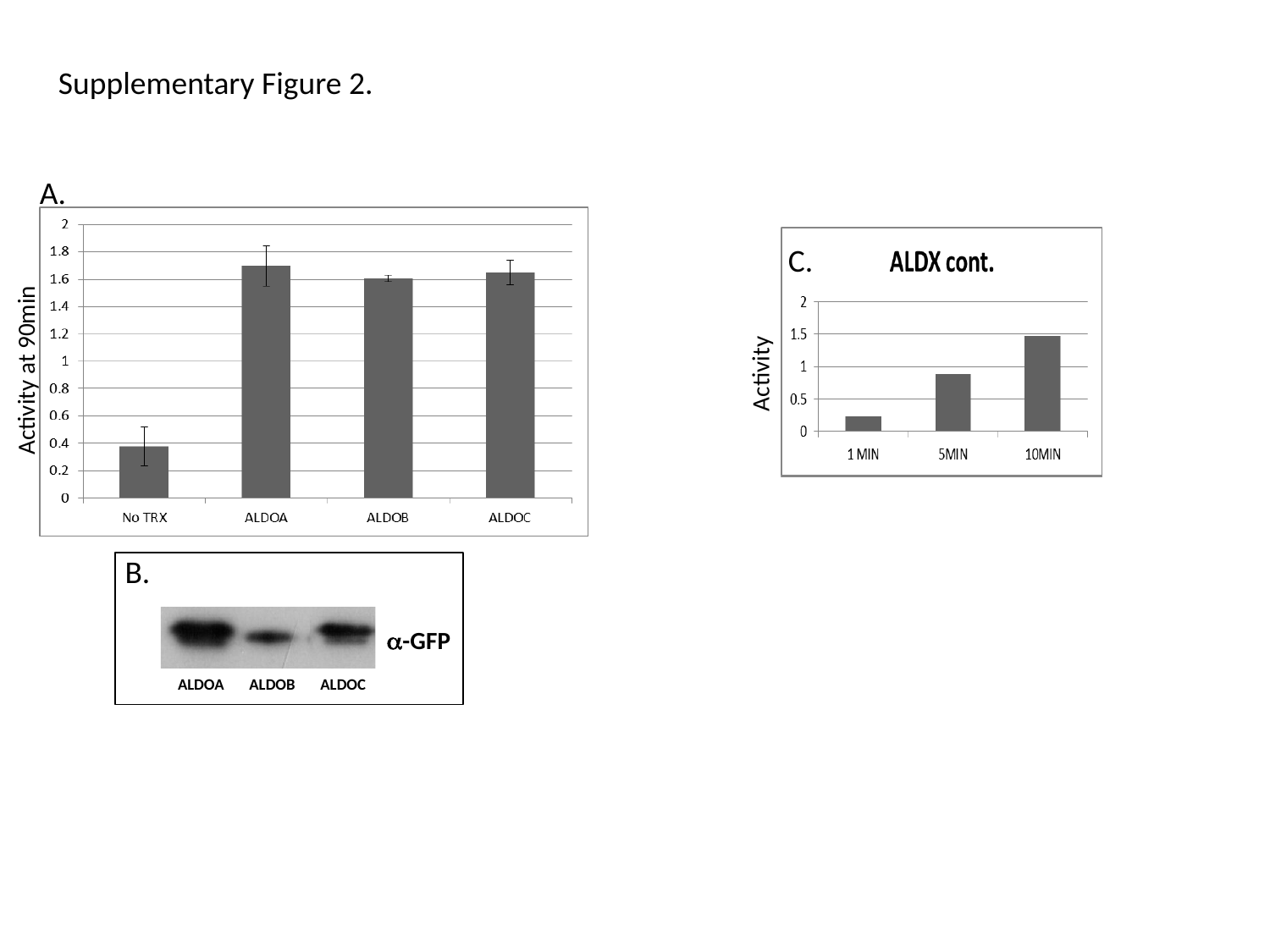

Supplementary Figure 2.
A.
 Activity
C.
 Activity at 90min
B.
a-GFP
ALDOA ALDOB ALDOC
