# Supplementary material for: Aldolase positively regulates of the canonical Wnt signaling pathway
Source: Mol Cancer. 2014 Jul 4;13:164. doi: 10.1186/1476-4598-13-164 (PMC4094682; doi:10.1186/1476-4598-13-164)
Supplement: Additional file 2 — Enzymatic activity of wild type and mutated aldolases. HEK293T cells were transfected with the indicated plasmids and subjected to immunoprecipitation using mouse anti-GFP antibody. Enriched aldolase proteins were then incubated with Fructose-1,6-bisphosfate and Hydrazine sulfate for 90 min. in order to measure enzymatic activity. A. Activity of the different aldolase isoforms compared to non-transfected extracts. B. HEK293T cells lysates were harvested as described and subjected to Western blot analysis to detect the transfected ALDO proteins. C. Purified ALDX from rabbit muscle (Sigma) was used as positive control. [file 1476-4598-13-164-S2.docx]

**Supplementary information** - Aldolase Positively Regulates of the Canonical Wnt Signaling Pathway

**Supplementary Results:**

Our results show that the three different aldolase isoforms increase Wnt signaling levels. In order to assess the exact mechanism of their action we have tested their enzymatic activity based on the method described in Jagannathan *et al*. 1956 ([Jagannathan, Singh and Damodaran 1956](#_ENREF_1)) . GFP-ALDO constructs were expressed and immunoprecipitated from HEK293T cells using anti-GFP antibodies to diminish the effect of endogenous aldolase (SFig. 2B). The enriched protein fraction was incubated with the aldolase substrate and activity was measured (SFig. 2A). As seen in figure S2A all three isoforms sustain enzymatic activity although somewhat lower compared to the commercial purified ALDX (SFig. 2C).

**Supplementary Legends:**

**SFig. 1.** TINA analysis of three separated experiments where the effect of GFP, GFP-ALDOB and GFP-ALDOC over-expression on different components of the Wnt cascade was measured. A representative blot is shown in Fig. 1C.

**SFig. 2. Enzymatic activity of wild type and mutated aldolases.** HEK293T cells were transfected with the indicated plasmids and subjected to immunoprecipitation using mouse anti-GFP antibody. Enriched aldolase proteins were then incubated with Fructose-1,6-bisphosfate and Hydrazine sulfate for 90 min. in order to measure enzymatic activity ([Jagannathan et al. 1956](#_ENREF_1)). **A.** Activity of the different aldolase isoforms compared to non-transfected extracts. B. HEK293T cells lysates were harvested as described and subjected to Western blot analysis to detect the transfected ALDO proteins. C. Purified ALDX from rabbit muscle (Sigma) was used as positive control.

**Supplementary Methods:**

**Aldolase activity.** HEK293T cells grown in 10 mm plates were transfected as previously described with GFP-ALDOB, GFP-ALDOC or an empty GFP vector. Forty-eight hours later the cells were subjected to immunoprecipitation as mentioned in the manuscript, using the mouse anti-GFP antibody. Enriched aldolase proteins were then incubated with Fructose-1,6-bisphosfate and Hydrazine sulfate (Sigma) for 90 min at room temp. in order to measure enzymatic activity ([Jagannathan et al. 1956](#_ENREF_1)). Purified ALDX from rabbit muscle (Sigma) was used as positive control.

**Reference:**

Jagannathan, V., K. Singh & M. Damodaran (1956) Carbohydrate metabolism in citric acid fermentation. 4. Purification and properties of aldolase from Aspergillus niger. *Biochem J,* 63**,** 94-105.
